# Preventable Deaths

**Published:** 1917-05

**Authors:** 


### Preventable Deaths.

Unless the physicians of the country take some kind of concerted action looking to the prevention of disease, it is likely that the government will take more rigid steps to prevent this kind of public waste. It is even possible that the government, provoked by the apparent indifference of a large part of the medical profession, may adopt the rather socialistic policy of health insurance and may undertake to care for the health of its people. More unlikely things have happened.

This observation is caused by the statement just read, that in 1913 there were over 350,000 deaths in this country from preventable diseases, or nearly one thousand for every day in the year. Every life is a cash asset to the government, at least in this country, and one that can be closely estimated in dollars. The government is a distinct loser by these 350,000 deaths a year that could be stopped with proper effort. It is fairly safe to say that if this great waste goes on much longer the government will decide that from both an economic and humanitarian standpoint it will pay it to conserve the lives of the people, and that will logically lead to requiring the people to pay the bills through some form of health insurance which will carry with it needed medical and surgical attention.

In such event, what would become of the doctors and surgeons of the country who do not become government officials with about the same compensation and standing of the average government employe? Doctors who are indifferent to stopping preventable diseases should perk up a bit and see the trend of affairs if they would save themselves.

